# Comparing Job Stress and Well‐Being Between Hospital and Long‐Term Care Nurses: A Cross‐Sectional Study in Taiwan

**DOI:** 10.1155/jonm/4735204

**Published:** 2026-05-19

**Authors:** Wen-Jye Shy, Kai-Lin Liang, Yi-Chun Hung

**Affiliations:** ^1^ Department of Nursing, Taichung Veterans General Hospital Puli Branch, Puli, Taiwan; ^2^ Aging Health and Long‐Term Care Management for Indigenous Undergraduate Program, National Chi Nan University, Puli, Taiwan, ncnu.edu.tw; ^3^ Department of Population Health Sciences, National Health Research Institutes, Zhunan, Taiwan, nhri.org.tw

**Keywords:** hospital, job demands–resources model, job stress, long-term care, nurses, nursing management, Taiwan, well-being

## Abstract

**Background:**

As population aging intensifies, the nursing workforce faces increasing pressure across hospital and long‐term care (LTC) sectors. Understanding how different work environments influence job stress and well‐being is essential for improving workforce sustainability and care quality.

**Objectives:**

This study aimed to compare job stress and well‐being between hospital and LTC nurses in Taiwan and to identify key demographic, occupational, and psychosocial factors associated with well‐being.

**Methods:**

Design and methods were guided by the Job Demands–Resources (JD–R) framework. A cross‐sectional design was conducted with 275 registered nurses recruited from hospitals and LTC institutions. Data were collected using a structured questionnaire that included the Nursing Staff Stress Scale and the WHO‐5 Well‐Being Index. Descriptive statistics, independent *t*‐tests, Pearson correlations, and multiple regression analyses were performed.

**Results:**

Hospital nurses reported significantly higher job stress, particularly in the domains of personal response, work concern, and inability to complete private work, while LTC nurses demonstrated higher levels of well‐being. Well‐being was negatively correlated with job stress. Regression analysis revealed that shorter working hours, fewer delayed off‐duty occurrences, higher income, being married, and higher education were significantly associated with better well‐being. The final model explained 37% of the variance in well‐being.

**Conclusions:**

Substantial differences in stress and well‐being exist between hospital and LTC nurses. Addressing workload, improving organizational support, and promoting balanced work conditions are essential for sustaining the mental health and stability of the nursing workforce. These findings provide actionable insights for nursing managers and policymakers aiming to design evidence‐based interventions that enhance workforce sustainability and psychological health. Practical strategies—such as reducing excessive working hours, minimizing delayed off‐duty time, and strengthening leadership support and flexible scheduling systems—are essential to enhance nurses’ well‐being and workforce sustainability.

## 1. Introduction

The global nursing workforce is under growing strain due to demographic shifts, workforce shortages, and increasing job demands. According to the World Health Organization, the world faces a projected shortage of 5.7 million nurses by 2030, with Asia particularly affected due to aging populations and rising long‐term care (LTC) needs [1]. This shortage has intensified workloads, increased overtime, and contributed to higher burnout and turnover intention among nurses [2]. Such workforce challenges directly impact patient safety and care quality, emphasizing the need to understand how different healthcare environments affect nurses’ well‐being and stress levels [3].

### 1.1. Job Stress, Well‐Being, and Work–Life Balance (WLB)

Nurses are exposed to multiple stressors, including long working hours, emotional labor, and heavy patient loads, which are associated with reduced well‐being and increased turnover intention [4]. The Job Demands–Resources (JD–R) model provides a comprehensive framework for understanding how these stressors operate. It posits that excessive job demands—such as time pressure, workload, and emotional strain—can lead to exhaustion, whereas adequate job resources, such as autonomy, leadership support, and social support, promote engagement and buffer stress [5–8].

Recent empirical studies confirm that job demands were significantly associated with burnout and turnover, while supportive work environments enhance resilience, satisfaction, and subjective well‐being (SWB) [9, 10]. For example, a recent meta‐analysis found that the global prevalence of turnover intention among emergency nurses was approximately 45%, with Asian nurses—especially those under 35 years—reporting the highest rates [2]. Moreover, a Taiwanese study highlighted that exposure to workplace violence (WV) was significantly associated with turnover intention, with social support and job control playing an important role in this relationship [11]. Collectively, these findings emphasize that high job demands, limited psychological safety, and insufficient organizational support remain key contributors to nurse burnout and attrition, whereas supportive and resource‐rich environments promote well‐being and workforce stability.

The JD–R framework has also been widely applied to understand WLB among healthcare professionals. Long working hours and low job control have consistently been associated with burnout, whereas supportive work environments and better WLB contribute to improved well‐being [5, 6]. Recent international evidence further indicates that poor WLB is strongly associated with lower SWB and poorer perceived health among healthcare workers. These findings highlight that nurses’ well‐being is not merely an individual issue but a systemic outcome shaped by organizational conditions and work design.

Cross‐national evidence indicates that quality of work life (QoWL) and SWB among healthcare professionals are shaped by workload, institutional support, and structural factors such as staffing ratios and professional development access [12, 13]. Although these patterns recur across cultural contexts, their manifestation varies by organizational setting and national policy environment, underscoring the need for context‐specific inquiry.

### 1.2. Hospital Versus Long‐Term Care (LTC) Work Contexts

The stressors facing hospital and LTC nurses differ markedly in both intensity and nature. Hospital nurses manage acute clinical demands, time‐sensitive procedures, and heavy documentation within hierarchical and technology‐driven systems. In contrast, LTC nurses and care aides deliver relationship‐based, continuous care for frail older adults, facing chronic workloads, emotional labor, and limited institutional resources [12, 14]. Despite these pressures, LTC workers often report stronger relational continuity, greater autonomy, and deeper meaning derived from sustained resident interaction [15, 16].

Job satisfaction determinants vary substantially between acute and residential care settings. Squires et al. [15] identified empowerment, autonomy, and adequate facility resources as the strongest predictors of satisfaction among LTC aides, whereas workload intensity and weak administrative support were key sources of dissatisfaction. Andersen [16] further emphasized that although LTC staff frequently experience heavy workloads and limited training, meaningful relationships and peer collaboration help sustain their morale and professional identity. These findings highlight that emotional fulfillment and social connection serve as intrinsic resources buffering burnout in LTC environments.

Conversely, hospital nurses’ well‐being is strongly influenced by leadership, staffing adequacy, and opportunities for advancement. Crocker [17] found that effective leadership and clear growth pathways promote job satisfaction. Although LTC work offers more stable schedules and emotionally meaningful roles, it is often associated with lower pay, fewer career opportunities, and persistent staff shortages [13]. A systematic review confirmed that such structural inequities intensify emotional exhaustion and contribute to workforce attrition [18].

The JD–R and Job Demand–Control–Support (JDCS) models provide a valuable framework for interpreting these contextual disparities. High workload and limited control elevate stress, whereas autonomy, peer cohesion, and organizational recognition act as protective factors [15, 19]. Within Taiwan’s rapidly expanding LTC sector, these dynamics are particularly salient. Although many LTC nurses demonstrate strong intrinsic motivation and affective commitment, they continue to face wage gaps and inadequate professional development compared with hospital peers [13, 16, 20].

Understanding how these contrasting work contexts—acute hospitals emphasizing task efficiency versus LTC settings prioritizing relational continuity—shape nurses’ stress and well‐being is crucial. Evidence suggests that enhancing leadership quality, professional recognition, and staffing support can reduce burnout and improve retention across both care settings. Tailored policies that balance emotional and structural resources are therefore essential for sustaining a resilient and satisfied nursing workforce in an aging society.

### 1.3. Integrating Global and Local Perspectives

Recent global reviews highlight that interventions focused on leadership, psychological capital, and digital monitoring tools—such as the Quan Well‐Being Index—can enhance well‐being by integrating mental, physical, and social domains [21, 22]. Additionally, autonomy, peer support, and fair workload distribution have been shown to protect nurses against psychological strain and turnover intention [6].

Grounded in the JD–R and QoWL frameworks, this study addresses a critical empirical gap in the nursing workforce literature. While prior studies have extensively documented burnout and turnover intention among hospital‐based nurses, comparatively little attention has been paid to how the distinct organizational environment of Taiwan’s rapidly expanding LTC sector—shaped by the *Long-Term Care 2.0* policy—differentially influences nurses’ SWB [9, 23, 24]. Most existing comparative work either pools heterogeneous settings without controlling for policy context or focuses on hospital nurses alone, thereby obscuring how structural and relational resources unique to LTC work may confer well‐being advantages.

By directly comparing hospital and LTC nurses within the same cultural and regulatory context, this study offers a theoretically grounded and policy‐relevant test of JD–R processes in an aging Asian society. The findings aim to inform nursing management strategies and workforce policies that sustain psychological health across both care sectors.

**FIGURE 1:**
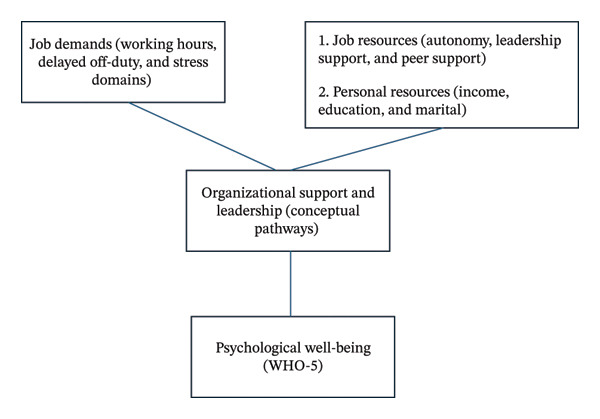
Conceptual framework based on the Job Demands–Resources (JD–R) and Job Demand–Control–Support (JDCS) models. Job demands (e.g., working hours, delayed off‐duty, and stress domains) are expected to negatively influence well‐being. In contrast, job resources (e.g., autonomy, leadership support, and peer support) and personal resources (e.g., income, education, and marital status) are expected to buffer the impact of job demands and enhance well‐being. Organizational support and leadership are conceptualized as key contextual factors that may facilitate resilience and work engagement, although no formal mediation is tested in this study.

The conceptual framework (Figure 1) illustrates the hypothesized relationships derived from the JD–R and JDCS models. Job demands (e.g., working hours, delayed off‐duty, and stress domains) are expected to negatively affect well‐being, whereas job and personal resources (e.g., income, education, marital status, and autonomy) are anticipated to buffer stress and enhance well‐being. Organizational support and leadership are proposed as potential mediating factors that foster resilience and engagement among nurses.

Accordingly, this study seeks to elucidate how work environment characteristics and psychosocial resources jointly influence nurses’ well‐being, providing evidence for strategies to reduce workload, enhance organizational support, and improve QoWL. The results aim to inform workforce policies that foster sustainable nursing practice and promote psychological health and resilience across both hospital and LTC sectors in Taiwan.

## 2. Methods

### 2.1. Study Design

This study employed a cross‐sectional, comparative design to examine differences in job stress and well‐being between hospital and LTC nurses in Taiwan. The research followed the STROBE reporting guidelines for observational studies to ensure methodological rigor.

### 2.2. Study Context

Taiwan’s healthcare system consists of a highly developed hospital sector and a rapidly expanding LTC system under the Long‐Term Care 2.0 policy. Hospitals are characterized by high patient acuity, hierarchical structures, and intensive workloads, while LTC services include institutional, community‐based, and home‐care settings with greater emphasis on continuity of care and relational interaction.

Despite policy efforts to expand LTC services, workforce shortages, lower wage levels, and limited career advancement opportunities remain challenges in the LTC sector. These contextual differences are important for interpreting variations in job stress and well‐being between hospital and LTC nurses in Taiwan.

### 2.3. Participants and Sampling

Participants were licensed registered nurses currently employed in either hospital or LTC settings. The hospital group included nurses working in acute care and medical‐surgical wards, while the LTC group comprised those working in institutional, community‐based, or home‐care units. Nurses holding administrative roles such as head nurses, supervisors, or directors were excluded to ensure comparability of frontline work experiences.

A purposive sampling method was used to recruit participants from hospitals and LTC facilities across northern, central, and southern Taiwan. A total of 275 valid responses were collected, including 148 hospital nurses (53.8%) and 127 LTC nurses (46.2%). All participants provided informed consent before completing the survey.

A post hoc power analysis using G^∗^Power 3.1 indicated that the sample size of 275 provided sufficient power (0.88) to detect a medium effect size (*f* ^2^ = 0.15) at a significance level of 0.05. This suggests adequate statistical power for regression analyses.

A total of 344 questionnaires were distributed, of which 275 were returned, yielding a response rate of 79.94%. After excluding incomplete responses, all 275 questionnaires were considered valid and included in the final analysis.

Participants were eligible for inclusion if they were (1) licensed registered nurses in Taiwan and (2) currently employed in frontline clinical roles in either acute hospital wards or LTC settings. Exclusion criteria were applied to (1) nurses holding administrative or managerial roles (e.g., head nurses, supervisors, or directors) to ensure the data captured the experiences of frontline clinical practice and (2) nurses with less than 3 months of tenure in their current facility to ensure they had sufficient exposure to their specific work environment.

### 2.4. Measures

#### 2.4.1. Demographic and Work‐Related Variables

Information was collected on participants’ age, gender, marital status, education level, years of service, monthly income, daily working hours, and frequency of delayed off‐duty time (i.e., staying beyond scheduled working hours due to unfinished tasks or workload demands).

#### 2.4.2. Taiwan Nursing Staff Stress Scale (TNSS)

Job stress was measured using the 24‐item TNSS developed for Taiwanese nurses. The scale includes four dimensions: (a) Personal Response; (b) Work Concern; (c) Competence; and (d) Inability to Complete Private Work. Inability to complete private work refers to the extent to which job demands interfere with nurses’ ability to attend to personal life responsibilities, such as family obligations, rest, and daily activities. Items were rated on a five‐point Likert scale (1 = *strongly disagree* to 5 = *strongly agree*), with higher scores indicating greater perceived stress. The total scale demonstrated excellent internal consistency in this study (Cronbach’s *α* = 0.91), with subscale *α* values ranging from 0.82 to 0.88.

#### 2.4.3. WHO‐5 Well‐Being Index

SWB was assessed using the World Health Organization Five Well‐Being Index (WHO‐5), a five‐item measure that evaluates positive mood, relaxation, vitality, and general life satisfaction. Scores range from 0 to 25, with higher scores reflecting better well‐being. Cronbach’s *α* in this study was 0.89, indicating high reliability.

### 2.5. Data Collection Procedure

Data were collected between January and March 2025. After obtaining ethical approval, research assistants distributed printed questionnaires to eligible nurses through institutional channels. Participation was voluntary and anonymous, and respondents returned completed questionnaires in sealed envelopes to ensure confidentiality. Each participant received a small token of appreciation upon survey completion.

To minimize potential response bias, data were collected from diverse geographical regions (northern, central, and southern Taiwan), and anonymity was strictly maintained. A Harman’s single‐factor test revealed that the first factor accounted for 28.6% of the variance, indicating that common method bias was not a significant concern.

### 2.6. Data Analysis

All analyses were performed using SPSS Version 26.0 (IBM Corp., Armonk, NY, USA). Descriptive statistics (means, standard deviations, and percentages) were calculated to summarize demographic and work characteristics. Independent‐sample *t*‐tests and one‐way ANOVA were applied to compare group means between hospital and LTC nurses. Chi‐square tests were used for categorical variable comparisons.

Pearson correlation coefficients were computed to examine relationships between job stress dimensions and well‐being. Multiple regression analysis was conducted to identify factors associated with well‐being. All demographic, occupational, and job stress variables were entered simultaneously into the model to estimate their independent associations with well‐being. Statistical significance was set at *p* < 0.05 for all analyses.

### 2.7. Ethical Considerations

This study was reviewed and approved by the Institutional Review Board of Jen‐Ai Hospital Dali Branch, Jen‐Ai Medical Foundation (IRB No. 202500024B0). The research team obtained the official certificate of clinical research consent following ethical approval. All participants were informed about the purpose of the study, the voluntary nature of participation, and their right to withdraw at any stage without penalty. Data were anonymized prior to analysis to ensure confidentiality, and all procedures conformed to the ethical principles outlined in the Declaration of Helsinki.

## 3. Results

### 3.1. Participant Characteristics

A total of 275 nurses participated in this study, including 148 (53.8%) from hospitals and 127 (46.2%) from LTC settings. The majority were female (92.0%), with a mean age of 36.4 ± 7.8 years. Hospital nurses had significantly longer working hours (9.7 ± 1.3 h/day) and more frequent delayed off‐duty times (i.e., staying beyond scheduled working hours due to unfinished tasks; 3.1 ± 1.9 times/week) than LTC nurses (8.3 ± 1.2 h/day, 1.5 ± 1.3 times/week, both *p* < 0.001). LTC nurses tended to have a slightly higher mean age, longer years of service, and lower monthly income (NT$46,900 ± 8300) compared with hospital nurses (NT$52,400 ± 9200). Marital status and education levels were not significantly different between groups (Table 1).

**TABLE 1:** Demographic and work‐related characteristics of participants (*N* = 275).

Variable	Total (*N* = 275)	Hospital (*n* = 148)	LTC (*n* = 127)	*χ* ^2^/*t*	*p*‐value
Age (years), mean ± SD	36.4 ± 7.8	35.7 ± 7.4	37.2 ± 8.1	1.86	0.064
Gender (female), *n* (%)	253 (92.0)	138 (93.2)	115 (90.6)	0.57	0.450
Marital status (married), *n* (%)	179 (65.1)	89 (60.1)	90 (70.9)	3.72	0.054
Education (bachelor’s or higher), *n* (%)	227 (82.5)	126 (85.1)	101 (79.5)	1.46	0.228
Monthly income (NT$), mean ± SD	49,800 ± 8900	52,400 ± 9200	46,900 ± 8300	4.92	< 0.001
Daily working hours, mean ± SD	9.1 ± 1.4	9.7 ± 1.3	8.3 ± 1.2	8.42	< 0.001
Delayed off‐duty (times/week), mean ± SD	2.4 ± 1.8	3.1 ± 1.9	1.5 ± 1.3	7.03	< 0.001
Years of service, mean ± SD	11.2 ± 6.5	10.8 ± 6.2	11.7 ± 6.9	0.97	0.332

*Note:* Delayed off‐duty refers to staying beyond scheduled working hours due to unfinished tasks or workload demands. This measure reflects workload‐related overtime rather than scheduled shift extensions.

### 3.2. Differences in Job Stress and Well‐Being

Table 2 presents the comparison of job stress dimensions between hospital and LTC nurses. Hospital nurses reported significantly higher job stress in personal response (*M* = 3.98 vs. 3.41, *p* < 0.001), work concern (*M* = 4.12 vs. 3.52, *p* < 0.001), and inability to complete private work (i.e., interference of job demands with personal life responsibilities; *M* = 3.76 vs. 3.22, *p* < 0.001). No significant difference was found in the competence domain. Overall job stress was markedly higher among hospital nurses (*M* = 3.83 ± 0.56) than LTC nurses (*M* = 3.37 ± 0.60, *p* < 0.001).

**TABLE 2:** Comparison of job stress scores between hospital and long‐term care nurses.

Stress dimension	Hospital (mean ± SD)	LTC (mean ± SD)	*t*	*p* *-*value
Personal response	3.98 ± 0.62	3.41 ± 0.71	6.74	< 0.001
Work concern	4.12 ± 0.57	3.52 ± 0.68	7.21	< 0.001
Competence	3.45 ± 0.69	3.32 ± 0.71	1.46	0.146
Inability to complete private work	3.76 ± 0.64	3.22 ± 0.72	5.97	< 0.001
**Overall stress score**	**3.83 ± 0.56**	**3.37 ± 0.60**	**7.18**	**< 0.001**

*Note:* Bold values indicate the overall scores and statistically significant results (*p* < 0.05).

As shown in Table 3, LTC nurses demonstrated significantly greater well‐being scores (*M* = 17.8 ± 4.2) than hospital nurses (*M* = 14.5 ± 4.8, *p* < 0.001). Subgroup analysis (Table 4) further indicated that LTC nurses working in community‐based (*M* = 18.1 ± 3.9) and home‐care settings (*M* = 18.5 ± 4.1) reported significantly higher well‐being than those in residential LTC facilities (*M* = 16.2 ± 4.3, *p* = 0.019).

**TABLE 3:** Comparison of well‐being scores between hospital and long‐term care nurses.

Group	Mean ± SD	*t*	*p*‐value
Hospital nurses	14.5 ± 4.8		
LTC nurses	17.8 ± 4.2	5.46	< 0.001

*Note: Post hoc comparisons:* LTC nurses in community and home‐care settings showed significantly higher WHO‐5 scores than those in residential facilities (*p* < 0.05).

**TABLE 4:** Subgroup analysis of well‐being by LTC setting type (*n* = 127).

LTC type	Mean ± SD	*F*	*p*‐value	Post hoc
Residential LTC	16.2 ± 4.3	4.12	0.019	Community > residential^∗^
Community care	18.1 ± 3.9			
Home care	18.5 ± 4.1			

*Note:* Scheffé post hoc test, *p* < 0.05.

^∗^A significant difference between the groups (*p* < 0.05) based on the Scheffé post hoc test.

### 3.3. Correlation Between Job Stress and Well‐Being

Pearson’s correlation analyses revealed significant negative relationships between job stress dimensions and well‐being (Table 5). Among the stress domains, personal response (*r* = −0.48, *p* < 0.001) and inability to complete private work (*r* = −0.46, *p* < 0.001) exhibited the strongest inverse associations with well‐being, indicating that higher stress in these domains corresponded to lower psychological well‐being (PWB).

**TABLE 5:** Correlations between job stress dimensions and well‐being (*N* = 275).

Variable	Personal response	Work concern	Competence	Inability to complete private work	Well‐being
Personal response	1				
Work concern	0.62^∗∗^	1			
Competence	0.44^∗∗^	0.39^∗∗^	1		
Inability to complete private work	0.53^∗∗^	0.41^∗∗^	0.37^∗∗^	1	
**Well-being**	**−0.48**	**−0.42**	**−0.29**	**−0.46**	1

*Note:* Bold values indicate correlations between job stress dimensions and well‐being.

^∗∗^ *p* < 0.001.

### 3.4. Factors Associated With Well‐Being

Multiple regression analysis (Table 6), with all predictors entered simultaneously, identified key factors associated with well‐being. 95% confidence intervals (CIs) were calculated to provide additional information on the precision and magnitude of the estimates. Daily working hours were negatively associated with well‐being (*β* = −0.29, 95% CI: −0.43 to −0.15). Delayed off‐duty occurrences were also negatively associated with well‐being (*β* = −0.21, 95% CI: −0.37 to −0.05). In addition, being married (*β* = 0.18, 95% CI: 0.04–0.32), higher monthly income (*β* = 0.24, 95% CI: 0.10–0.38), and higher education level (*β* = 0.15, 95% CI: 0.03–0.27) were positively associated with well‐being. Stress dimensions—particularly personal response (*β* = −0.31, 95% CI: −0.49 to −0.13) and inability to complete private work (*β* = −0.28, 95% CI: −0.44 to −0.12)—were also significantly negatively associated with well‐being. The overall model explained 37% of the variance in well‐being (adjusted *R* ^2^ = 0.37, *p* < 0.001).

**TABLE 6:** Multiple regression analysis of factors associated with well‐being.

Predictor	*β*	SE	*t*	*p*‐value	95% CI
Daily working hours	−0.29	0.07	−4.38	< 0.001	(−0.43, −0.15)
Delayed off‐duty	−0.21	0.08	−3.12	0.005	(−0.37, −0.05)
Marital status (married = 1)	0.18	0.07	2.69	0.008	(0.04, 0.32)
Monthly income	0.24	0.07	3.16	0.003	(0.10, 0.38)
Education level	0.15	0.06	2.25	0.025	(0.03, 0.27)
Personal response (stress)	−0.31	0.09	−3.44	0.001	(−0.49, −0.13)
Inability to complete private work	−0.28	0.08	−3.25	0.002	(−0.44, −0.12)
**Adjusted** **R** ^2^	**0.37**				

*Note:* The bold value indicates the adjusted *R*_2_ of the regression model.

Collectively, these findings indicate that work environment, stress load, and personal factors jointly contribute to nurses’ well‐being. Hospital nurses’ longer working hours and greater task burden significantly undermine their PWB, whereas LTC settings—with a relatively lower workload and greater autonomy—are associated with more favorable well‐being outcomes.

## 4. Discussion

### 4.1. Key Findings and Interpretation

This study demonstrates significant contextual differences in job stress and well‐being between hospital and LTC nurses in Taiwan. Hospital nurses reported markedly higher job stress, particularly in the domains of personal response, work concern, and inability to complete private work, while LTC nurses exhibited greater well‐being. This dimension reflects work–life interference, indicating that high job demands may limit nurses’ ability to fulfill personal and family‐related responsibilities, thereby negatively affecting their PWB. These findings reinforce prior evidence that nurses in acute settings experience higher workloads and administrative burden than those in LTC or community‐based facilities [12, 14]. Consistent with Broetje et al. [24], such elevated job demands—especially workload and work‐life interference—represent critical risk factors for exhaustion and disengagement across healthcare systems. From a JD–R perspective, these findings support the health impairment process, in which sustained job demands deplete energy and lead to reduced well‐being. Within the JD–R framework, LTC nurses’ elevated well‐being should not be attributed solely to lower objective workload. More critically, *relational continuity*—the sustained, trust‐based interaction with older adult residents over time—functions as a potent emotional resource that generates affective rewards and a sense of occupational meaning. This resource dynamic is particularly salient in collectivist Asian societies, where interpersonal reciprocity and care‐giving identity are deeply intertwined with professional self‐efficacy [25]. Accordingly, even within structurally resource‐constrained LTC environments (lower wages and fewer advancement opportunities), relational continuity may compensate for material deficits by activating the JD–R motivational pathway—enhancing engagement and buffering against psychological strain.

The observed disparity between hospital and LTC nurses’ well‐being aligns with international patterns reported in Japan and South Korea, where hospital‐based nurses experience higher stress due to hierarchical work structures and acute patient acuity, whereas LTC nurses benefit from relational continuity and emotional reciprocity in older people care contexts. However, Taiwan’s LTC sector is characterized by lower pay and fewer advancement opportunities, underscoring the importance of policy‐level interventions that enhance both emotional and financial rewards.

Furthermore, according to the OECD [26] and WHO [1] workforce resilience frameworks, nurse well‐being is now considered a core indicator of health system performance. Thus, the present findings contribute to global efforts to establish evidence‐based policies that balance efficiency, equity, and psychological sustainability within the nursing workforce. In addition, system‐level strategies such as mandated staffing ratios and workload monitoring tools—such as resident assessment instruments (RAIs)—may support more accurate allocation of care resources and improve workforce sustainability. In this context, improving staffing adequacy, reducing excessive working hours, and strengthening workforce support mechanisms are essential to translate these findings into sustainable workforce policies [27]. These approaches can help align staffing levels with patient needs and reduce excessive workload burden on nurses, particularly in high‐demand care settings. This is consistent with prior evidence indicating that adequate nurse staffing levels are strongly associated with improved patient outcomes, job satisfaction, and reduced burnout [28, 29].

### 4.2. Theoretical Implications: JD–R, JDCS, and QoWL Perspectives

This study extends the JD–R framework by contextualizing it within the LTC sector of an aging Asian society, where relational continuity and affective commitment serve as critical resources. Unlike traditional JD–R applications that primarily emphasize structural and cognitive job resources, our findings highlight relational and emotional dimensions of care continuity as unique protective factors that enhance well‐being. This contextual expansion suggests that the JD–R model can be culturally adapted to account for collectivist values and interdependence inherent in Asian healthcare settings. Moreover, by integrating the QoWL perspective, previous research has shown that supportive work environments and positive organizational climates are associated with improved nurse outcomes, including job satisfaction and well‐being [30]. This is consistent with findings from recent systematic reviews indicating that job satisfaction among nurses is strongly influenced by factors such as workload, leadership support, and work environment, all of which are closely aligned with the job demands and resources framework [31, 32]. This research links job resources not only to psychological outcomes but also to broader workforce sustainability, emphasizing that emotional connectedness and perceived organizational support (POS) jointly constitute the foundation of well‐being among nurses.

Our findings align closely with the JD–R and JDCS models [5, 19], revealing that excessive working hours and delayed off‐duty time (high job demands) significantly undermine well‐being, whereas higher education, marital status, and income (job and personal resources) enhance resilience. Broetje et al. [24] highlighted the dual pathways of health impairment and motivation, emphasizing that demands such as overload and emotional labor reduce well‐being, while autonomy, leadership support, and fairness increase engagement. Our regression results (adjusted *R* ^2^ = 0.37) empirically substantiate these mechanisms, showing that personal and structural resources jointly were associated with well‐being.

Furthermore, this study conceptually highlights the potential role of POS in relation to PWB, although no formal mediation analysis was conducted. Therefore, interpretations regarding mediation should be made with caution, and future studies are encouraged to apply formal mediation models to examine these relationships. This is consistent with findings from Vietnam by Duong et al. [25], who demonstrated that POS enhances PWB, which in turn strengthens nurses’ intention to stay. These results suggest that organizational resources, particularly supportive management and perceived care from employers, are crucial in fostering nurses’ well‐being and retention. Beyond individual resilience, the sense of being valued and supported by one’s organization functions as a key protective factor against occupational stress and turnover in demanding healthcare environments. Future research employing structural equation modeling or longitudinal designs would provide stronger evidence for causal and mediating mechanisms.

### 4.3. Cross‐Cultural Insights and Gendered Context

Within East Asian contexts, cultural values emphasizing collectivism and endurance may shape how nurses perceive workload and emotional demands. Duong et al. [25] found that socio‐emotional reciprocity—employees feeling valued by their organization—enhances both commitment and psychological stability. Our findings align with this, as Taiwanese LTC nurses, though facing lower pay, may derive greater meaning from relationship‐centered care. Likewise, the predominance of women in the nursing workforce [22] highlights how gendered expectations intersect with occupational demands, often intensifying role conflict and emotional strain. This underscores the importance of gender‐sensitive workforce policies and mentorship models to address inequitable burdens.

### 4.4. Organizational and Policy Implications

The results suggest that interventions targeting workload reduction, schedule predictability, and enhancement of organizational support could meaningfully improve nurse well‐being. Duong et al. [25] emphasized that POS and organizational commitment mediate the link between support and retention, implying that supportive environments not only improve well‐being but also reduce turnover. Policymakers should therefore invest in leadership training, flexible work systems, and equitable pay structures to retain skilled nurses, particularly in LTC sectors where emotional labor is high but recognition remains low. Incorporating well‐being metrics into national quality indicators could further align institutional performance with the WHO’s call for health workforce resilience.

At the system level, regulatory approaches such as mandated staffing ratios and workload monitoring tools (e.g., RAIs) may help align care demands with available workforce capacity. These mechanisms can support more equitable distribution of workload and improve workforce sustainability, particularly in LTC settings where staffing shortages are more pronounced.

### 4.5. Limitations and Future Research

The cross‐sectional self‐report design limits the ability to infer causal relationships among job demands, resources, and well‐being. Future longitudinal or multiwave panel studies are recommended to capture temporal changes in stress and well‐being trajectories. Additionally, although efforts were made to include diverse geographic regions, the sample was limited to Taiwan and may not represent nurses in other cultural contexts. Comparative studies across East Asian and Western healthcare systems could further clarify how cultural values (e.g., collectivism vs. individualism) moderate JD–R processes.

Methodologically, incorporating multilevel modeling could help disentangle the effects of individual‐level and organizational‐level factors on well‐being. Future studies may also explore additional mediators, such as psychological capital, emotional intelligence, or perceived justice, to refine the understanding of how job resources enhance resilience and retention in diverse nursing environments.

Additionally, the LTC group included nurses from institutional, community‐based, and home‐care settings, which may differ substantially in job characteristics and autonomy. Although subgroup analyses were conducted, the aggregation of these diverse settings may have introduced heterogeneity. Future studies should further examine setting‐specific effects using more stratified or multilevel approaches.

Future research may also explore organizational‐level variables not directly measured in the present study—including leadership style, gender‐specific role demands, and digital well‐being assessment tools such as the Quan Well‐Being Index—which may serve as additional resources or moderating factors within the JD–R framework.

## 5. Conclusion

This study revealed clear differences in job stress and well‐being between hospital and LTC nurses in Taiwan. Hospital nurses experienced higher emotional strain, work concerns, and interference with personal life, while LTC nurses—especially those in community and home‐care settings—reported greater PWB. These results align with the JD–R and JDCS models, showing that long working hours and delayed off‐duty time undermine well‐being, whereas education, marital stability, and income act as protective resources.

Organizational and interpersonal supports, including autonomy and leadership, were key in buffering stress. Consistent with global findings, supportive and equitable leadership enhanced engagement and retention, while lack of recognition and work‐life imbalance contributed to burnout. Policy efforts should prioritize flexible scheduling, workload redistribution, and fair compensation, especially in the LTC sector. Integrating digital well‐being monitoring tools such as the Quan Well‐Being Index could further strengthen mental health management. Promoting nurses’ well‐being is essential not only for workforce sustainability but also for ensuring the quality of care in aging societies. These findings highlight the need for integrated organizational and policy‐level interventions to sustain a resilient nursing workforce.

## 6. Implications for Nursing Management

Based on the study findings, managerial interventions should be implemented across three interconnected levels:

Microlevel (individual):

• Develop resilience‐building programs and peer mentoring systems to enhance nurses’ coping skills and emotional regulation.
• Encourage self‐care routines and reflective practices through digital tools such as the Quan Well‐Being Index to promote self‐monitoring of stress and well‐being.

Mesolevel (organizational):

• Implement flexible and predictable scheduling systems to reduce delayed off‐duty occurrences and workload overload.
• Strengthen health‐promotive and transformational leadership training to cultivate supportive work environments.
• Introduce recognition and feedback mechanisms to enhance POS and fairness.

Macrolevel (policy):

• Incorporate nurse well‐being indicators into national accreditation standards and workforce performance metrics.
• Establish funding incentives for LTC institutions that provide continuing education and career advancement pathways.
• Align national workforce policies with the WHO’s and OECD’s frameworks for health system resilience and mental health promotion.

These strategies collectively underscore that nurse well‐being is not merely an individual issue but a systemic determinant of workforce stability and quality of care.

## Author Contributions

Wen‐Jye Shy conceived the study, formulated the research framework, and conducted literature and questionnaire collection.

Kai‐Lin Liang was responsible for writing the research architecture, drafting the manuscript, performing the core data analysis, and overall study coordination.

Yi‐Chun Hung provided essential support for statistical analysis.

## Funding

This research received no specific grant from any funding agency in the public, commercial, or not‐for‐profit sectors.

## Disclosure

All authors have read and agreed to the published version of the manuscript.

## Conflicts of Interest

The authors declare no conflicts of interest.

## Data Availability

Data sharing is not applicable to this article as no datasets were generated or analyzed during the current study.
